# Clinical Characteristics and Aqueous Humor Laboratory Analysis of Chinese Patients With Rubella Virus-Associated and Cytomegalovirus-Associated Fuchs Uveitis Syndrome

**DOI:** 10.3389/fmed.2020.610341

**Published:** 2020-12-18

**Authors:** Hao Kang, Han Bao, Yanhong Shi, Jing Feng, Weiqiang Yang, Yinzhang He, Hui Wang, Xiaofeng Hu, Yong Tao

**Affiliations:** Department of Ophthalmology, Beijing Chaoyang Hospital, Capital Medical University, Beijing, China

**Keywords:** fuchs uveitis syndrome (FUS), rubella virus (RV), cytomegalovirus (CMV), anterior uveitis, cytokines

## Abstract

**Purpose:** To describe and compare the clinical characteristics and laboratory analysis results of aqueous humor (AH) in fuchs uveitis syndrome (FUS) patients caused by rubella virus (RV) and cytomegalovirus (CMV).

**Methods:** A retrospective and observation-based study was performed on 32 patients with FUS. Etiologies, clinical characteristics, ocular complications, visual prognoses, inflammatory cytokines, and virus-specific antibodies in AH were compared.

**Results:** Among all the cases involved, 24 had RV FUS and 8 had CMV FUS. The mean age at diagnosis of FUS in the CMV group was older than that of the RV group (*P* = 0.031). The mean LogMAR best corrected visual acuity (BCVA) at initial presentation and at the final visit were both significantly higher in the CMV FUS group than those in the RV FUS group (*P* = 0.004, 0.047). The highest intraocular pressure (IOP) was significantly higher in the CMV group (*P* = 0.040). Consistent with elevated IOP, the CMV FUS patients were significantly more prone to developing glaucoma eventually than the RV FUS patients (*P* = 0.039). Vitreous opacity was found in 66.7% of the RV patients and 25.0% of the CMV patients (*P* = 0.038). The gender ratio, initial symptoms, presence and types of keratic precipitates, severity of anterior segment inflammation, iris lesions, and incidence of complicated cataract were similar between the two groups. There was no detectable difference of inflammatory cytokines in AH between RV FUS and CMV FUS.

**Conclusion:** The clinical manifestations and disease prognosis vary between CMV FUS and RV FUS. However, clinical differences are always not obvious enough for differential diagnosis. The laboratory AH analysis is necessary to identify the etiology, determine the therapeutic strategies, and assess the disease prognosis.

## Introduction

Fuchs uveitis syndrome (FUS), as a chronic, typically unilateral, usually asymptomatic mild inflammatory disorder, predominantly involves the anterior uvea and vitreous (1). Diagnosis of FUS is mainly based on clinical manifestations because the etiology remains obscure. Presumed pathogenic mechanisms of FUS include infections, autoimmune diseases, sympathetic dysfunction and hereditary factors (2). According to the theory of infection, the infection of rubella virus (RV), cytomegalovirus (CMV), herpes simplex virus (HSV), ocular toxoplasmosis, and other viruses have been implicated in the pathogenesis of FUS (2).

RV and CMV are the most commonly reported infectious etiologic agents of FUS (3–6). Several clinical studies have demonstrated that RV plays a pivotal role in the pathogenesis of FUS in Western patients (7–9). By contrast, FUS caused by CMV infection occurs more frequently in Asian countries. CMV infection accounts for 16–42% in Asian FUS cases of FUS, while Western FUS cases are predominantly associated with rubella virus rather than with CMV (5, 10, 11). As to different virus species of pathogenic microorganisms, there are some differences in clinical features among the patients from different etiological infections.

Therefore, we herein report a retrospective study of FUS patients with different viral etiologies in the Chinese population. It focuses on the clinical characteristics of viral FUS and on the laboratory analysis of aqueous humor (AH) that suggests a viral etiology and helps in differentiating one virus from another and in assessing the severity of intraocular inflammation.

## Materials and Methods

### Subjects

This retrospective and observation-based study included patients diagnosed with FUS at Beijing Chaoyang Hospital, which is affiliated with the Capital Medical University, from June 2018 to December 2019. Most of the patients were referred to our hospital by other hospitals.

The diagnosis of FUS was based primarily on the clinical characteristics: (i) absence of acute symptoms like severe eye pain; (ii) small to medium-size stellate keratic precipitates (KPs) diffusely spread the whole endothelial surface; (iii) a chronic low-grade inflammation in anterior chamber (AC); (iv) diffuse iris depigmentation with or without heterochromia; (v) lack of posterior synechiae. All the patients had received a complete checkup (Complete blood count, erythrocyte sedimentation rate, C-reactive protein, anti-streptolysin O titer, antinuclear antibody, rheumatoid factor, serum angiotensin-converting enzyme assay, tuberculin skin test, syphilis serology and chest X-ray) to exclude any other cause of uveitis (12).

The following anonymized data were reviewed from the patients' medical records: demographics, medical history, disease duration from onset to diagnosis, clinical presentations, clinical course, forms of treatment, ocular complications, vaccination history, imaging results and laboratory test results. Ophthalmic examinations, including determination of best-corrected visual acuity (BCVA), slit lamp examination for the anterior segment of the eye, gonioscopy, and ophthalmoscopy through the dilated pupil. BCVA was tested by using a Snellen chart, and BCVA was manifested by using the transformed logarithm of the minimum angel of resolution (LogMAR) values. The anatomical classification of uveitis and the grading scheme for the anterior chamber cells followed the criteria set by the International Uveitis Study Group (6, 13). Laser flare photometry was performed using the FC-2000 laser flare meter quantitatively to evaluate AC flare. By definition, glaucoma was identified as an intraocular pressure (IOP) of more than 21 mmHg and the occurrence of disc abnormalities, of visual field defects characteristic of glaucoma, or both. All eyes with elevated IOP were dealt with appropriately according to a standard protocol (including examinations and medications for glaucoma).

The institutional review board approved this retrospective study. AH and peripheral blood (PB) samples of all the patients were obtained only after informed consent and Ethics Committee approval. All procedures were performed according to the tenets of the Declaration of Helsinki.

In China, the RV vaccination program was offered to all children from 2010 in the combined rubella, mumps, and measles vaccination given at the age of 8–24 months. Therefore, all the patients born in China after 2010 and participating in the national vaccination program were regarded as vaccinated, while the patients born prior to 2010 were considered otherwise.

### Analysis of the AH Samples

AH samples (100–200 μl) were obtained by AC paracentesis. All of the obtained samples were promptly stored at 4°C and then brought to the laboratory for analysis. The AH samples were centrifuged at 350 g for 5 min. All the samples were then washed and resuspended with staining buffer. Serum samples were obtained at the time of AH tap and stored until processing.

Genomic DNA of the RV and CMV in the AH was measured using the rubella virus and cytomegalovirus nucleic acid assay kit (Shanghai ZJ Bio-Tech Co., Ltd.), and PCR was performed using the Multicolor Real-time PCR Detection System (LineGene 9600 Plus, BIOER TECHNOLOGY, Hangzhou). AH cytokines—IL-10, IL-6, IL-8, vascular endothelial growth factor (VEGF), and vascular cell adhesion molecules (VCAM)—were measured by means of CBA with BD FACSCantoII flow cytometry. Enzyme-linked immunosorbent assay (ELISA) for virus-specific antibody (IgG) detection in AH and serum sample was used to assess the Goldmann-Witmer coefficient (GWC) (Virion-Serion Biotechnology, Germany). We consider a GWC ratio >4 as confirmation of intraocular antibody production against the viral agent. For AH PCR, GWC and cytokine analysis, about 25 μl of AH is required for each test. Cytokine analyses were also performed on twenty normal eyes undergoing routine cataract surgery during the same period.

### Statistical Analysis

Statistical analysis was performed by using SPSS for Windows, version 24.0. Continuous variables were analyzed using a non-parametric test (Mann–Whitney *U*-test). The categorical data were analyzed by using the Chi-squared test or Fisher's exact test. The differences between the two groups were analyzed using the Kruskal–Wallis test and *post-hoc* tests with Bonferroni correction. *P* < 0.05 was considered statistically significant.

## Results

### Demographic Data and Clinical Characteristics

Thirty-two eyes of 32 patients (unilateral presentation in all the cases) who had either positive PCR or GWC for RV/CMV were included. Data were collected from 41 FUS patients, but later on nine patients were excluded, since six patients had no record of AH test and three patients had been lost in follow-up. No past history of immunodeficiency was reported for the patients included. The demographic data and clinical characteristics of the 24 RV-associated FUS and the eight CMV-associated FUS were compared (Table 1). All the patients were Chinese. None of the patients had undergone early childhood vaccination against RV. All FUS patients were born before the vaccination program was introduced in China.

**Table 1:** Demographics and clinical characteristics of the patients with RV FUS and CMV FUS.

	**RV FUS**	**CMV FUS**	***P*-value**
No. of patients	24	8	–
Age at the onset of the disease, mean ± SD	30.3 ± 11.1	37.5 ± 9.3	0.078^†^
Age at the diagnosis of FUS, mean ± SD	31.5 ± 10.8	41.3 ± 10.5	**0.031**^†^
Gender (male: female)	8: 16	4:4	0.433^‡^
Follow-up time (month), mean ± SD	14.2 ± 2.5	15.8 ± 4.1	0.346^†^
No. of recurrence, *n* (%)	0/24 (0%)	2/8 (25%)	0.056^‡^
**Symptoms**, ***n*** **(%)**			
Blurred or reduced vision	19/24 (79.2%)	7/8 (87.5%)	>0.99^‡^
Floaters	4/24 (16.7%)	2/8 (25.0%)	0.625^‡^
Eye pain	2/24 (8.3%)	1/8 (12.5%)	>0.99^‡^
Red eye	1/24 (4.2%)	0/8 (0%)	>0.99^‡^
Photophobia	1/24 (4.2%)	0/8 (0%)	>0.99^‡^
Anti-glaucoma drugs, *n* (%)	16/24 (66.7%)	7/8 (87.5%)	0.386^‡^
Topical steroid use, *n* (%)	22/24 (91.7%)	8/8 (100.0%)	>0.99^‡^

*RV, Rubella virus; CMV, Cytomegalovirus; FUS, Fuchs uveitis syndrome; SD, Standard deviation*.

† *Mann–Whitney U-test*.

‡ *Fisher's exact test*.

*Bold values indicate P < 0.05*.

Of the 32 patients, the median age at the onset of the disease was 30.3 years (range 9–55) in the RV-associated FUS group. The age at disease onset was older in the CMV-associated FUS group, but no significant difference between the two groups was found. The diagnosis of FUS was not always made immediately at presentation. The mean age at the diagnosis of FUS was 41.3 years (range 30–56) in the CMV-associated group and it was statistically older than that of the RV-associated FUS group (31.5 years, range 11–55, *P* = 0.031). The male-to-female ratio did not vary significantly between the two groups (*P* = 0.433). The mean follow-up time was 14.6 ± 2.9 months (range: 9–21), which did not vary significantly between the two groups. Disease recurrence was observed in two patients in the CMV group during follow-up, but not in the RV group during follow-up. The initial ocular symptoms that prompted the patients to visit the ophthalmology clinic were, in descending order, blurred vision or reduced vision, floaters, eye pain, red eye, and photophobia. There was no statistically significant difference in the initial symptoms between the two groups.

All the patients had been treated in other hospitals before their referral to this uveitis study center. FUS was diagnosed previously in nine patients by the referring ophthalmologists in the RV group, but only one patient in the CMV group had a prior diagnosis of FUS. Previous increased IOP and use of anti-glaucoma drugs during the chronic course were frequent: in 16 (66.7%) of the 24 RV FUS patients, in 7 (87.5%) of the eight CMV FUS patients (*P* = 0.386). All the patients in the two groups had been treated with topical steroid before first observation in this center. In the CMV FUS group, 3 (37.5%) of the eight patients were given a course of systemic corticosteroid therapy. Routine phacoemulsification and intraocular lens implantation was performed in three eyes (9.4%) in this center. BCVA at the end of the follow-up was ≥0.8 in two patients. In another case, no improvement in BCVA was observed after surgery due to secondary glaucoma preoperatively. Trabeculectomy was performed in two patients in the CMV FUS group.

**Figure 1:**
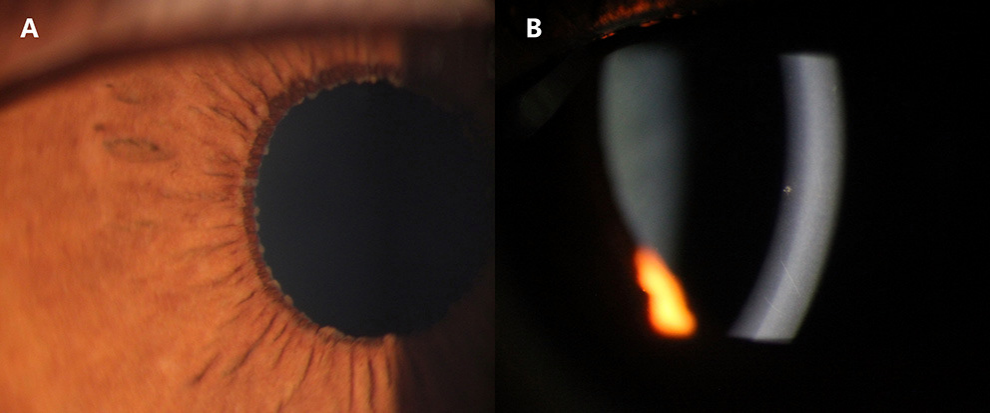
Slit-lamp photographs of two Cytomegalovirus (CMV)-associated Fuchs uveitis syndrome (FUS) patients. Iris Koeppe's nodule of a 39-year-old male patient **(A)**. Coin-shaped Keratic precipitates (KPs) of a 32-year-old female patient **(B)**.

All the patients underwent a visual functional examination during their first presentation and the follow-up period (Table 2). The mean LogMAR BCVA at the initial presentation and at the final visit were both significantly higher in the CMV FUS group than those in the RV FUS group (*P* = 0.004, 0.047). The highest IOP in the CMV-FUS group (23.5 ± 9.5 mmHg) was significantly higher than the highest IOP in the RV-FUS group (17.1 ± 6.8 mmHg) (*P* = 0.040). KPs were usually stellate and fine to medium-sized in morphology in most cases and were diffusely distributed on the whole endothelial surface in most cases. The types of KPs did not differ remarkably between the two groups. Mild to moderate AC activities were observed in both groups. Mean laser flare photometry values in the two groups of patients were 9.70 ± 6.32 and 10.65 ± 4.10 photon counts per millisecond, respectively (*P* = 0.591). There was no significant difference between the RV and CMV groups in the AC inflammatory response. Iris depigmentation or atrophy occurred in four eyes (12.5%) and iris heterochromia was observed in 10 eyes (31.3%). Iris nodules were observed in nine eyes (28.1%) (Koeppe nodules in 8 and Busacca nodules in 1) (Figure 1). No posterior synechiae were observed in all the involved eyes. Complicated cataract at presentation was observed in 24 eyes (75.0%), of which 19 showed a posterior subcapsular opacity. The patients with cataract had varying degrees of visual impairment. RV FUS patients had vitreous opacity more frequently than CMV FUS patients and the difference between them was statistically significant (*P* = 0.038). No significant difference in vitreous cells between the two groups was found (*P* = 0.106). Consistent with elevated IOP, the CMV FUS group was significantly more likely to reach the glaucomatous optic neuropathy over time than the RV FUS group (*P* = 0.039). No choroidalretina scars and cystoid macular edema were found in the fundus examination of the FUS patients in both groups. In the RV FUS group, two patients had fluorescein angiography performed with vascular leakage but without staining of the disc.

**Table 2:** Clinical manifestations and complications at presentation of the patients with RV FUS and CMV FUS.

	**RV FUS**	**CMV FUS**	***P*-value**
	**(*n* = 24)**	**(*n* = 8)**	
LogMAR initial BCVA, mean ± SD	0.30 ± 0.19	0.75 ± 0.41	**0.004**^†^
LogMAR final BCVA, mean ± SD	0.26 ± 0.16	0.70 ± 0.63	**0.047**^†^
Highest IOP (mmHg), mean ± SD	17.1 ± 6.8	23.5 ± 9.5	**0.040**^†^
Corneal endothelial lesions, *n* (%)	0 (0%)	1 (12.5%)	0.250
**KPs**, ***n*** **(%)**			
Stellate	17/24 (70.8%)	5/8 (62.5%)	0.681^‡^
Fine	5/24 (20.8%)	0/8 (0%)	0.296^‡^
Medium	0/24 (0%)	2/8 (25.0%)	0.056^‡^
Mutton fat	2/24 (8.3%)	1/8 (12.5%)	>0.99^‡^
Coin	0/24 (0%)	1/8 (12.5%)	0.250^‡^
Pigmented	0/24 (0%)	1/8 (12.5%)	0.250^‡^
Aqueous flare, mean ± SD	9.70 ± 6.32	10.65 ± 4.10	0.591^†^
**Anterior chamber cells**, ***n*** **(%)**			
0–1	13/24 (54.2%)	4/8 (50.0%)	>0.99^‡^
≥1	2/24 (8.3%)	1/8 (12.5%)	>0.99^‡^
**Iris atrophy**, ***n*** **(%)**			
Depigmentation/ Atrophy	2/24 (8.3%)	2/8 (25.0%)	0.254^‡^
Heterochromia	8/24 (33.3%)	2/8 (25.0%)	>0.99^‡^
**Iris nodules**, ***n*** **(%)**			
Koeppe nodules	7/24 (29.2%)	1/8 (12.5%)	0.642^‡^
Busacca nodules	1/24 (4.2%)	0/8 (0%)	>0.99^‡^
Iris posterior synechiae, *n* (%)	0 (0%)	0 (0%)	–
Cataract, *n* (%)	17/24 (70.8%)	7/8 (87.5%)	0.642^‡^
Vitreous opacity, *n* (%)	17/24 (66.7%)	2/8 (25.0%)	**0.038**^‡^
Vitreous cell, *n* (%)	15/24 (62.5%)	2/8 (25.0%)	0.106^‡^
Glaucoma, *n* (%)	1/24 (4.2%)	3/8 (37.5%)	**0.039**^‡^
Chorioretinal scars, *n* (%)	0 (0%)	0 (0%)	–

*RV, Rubella virus; CMV, Cytomegalovirus; FUS, Fuchs uveitis syndrome; BCVA, Best corrected visual acuity; IOP, Intraocular pressure; KPs, Keratic precipitates; SD, Standard deviation*.

† *Mann–Whitney U-test*.

‡ *Fisher's exact test*.

*Bold values indicate P < 0.05*.

### Comparative Laboratory Analysis of AH in RV FUS and CMV FUS Patients

The expression of cytokines in the AH of the RV-associated and CMV-associated FUS patients was summarized in Table 3. Compared with noninflammatory controls, inflammatory cytokines in AH were elevated in both FUS groups. However, no discernible differences between the two groups were observed among IL-10, IL-6, IL-8, VEGF, and VCAM. The detection results of inflammatory cytokines in AH were consistent with the AC inflammatory reaction in clinical manifestations. Both groups showed mild AC inflammation, but there was no significant difference between the two groups.

**Table 3:** Cytokines levels in the AH of RV FUS, CMV FUS, and cataract patients.

	**RV FUS**	**CMV FUS**	**Cataract**	***P*^†^**	*P*1^*^	*P*2^*^	*P*3^*^
	**(*n* = 24)**	**(*n* = 8)**	**(*n* = 20)**		**RV and CMV**	**RV and C**	**CMV and C**
IL-10 mean ± SD (pg/ml)	5.12 ± 4.59	8.07 ± 5.30	1.83 ± 1.49	**0.017**	0.280	**0.028**	**0.002**
IL-6 mean ± SD (pg/ml)	57.84 ± 39.59	43.23 ± 46.45	18.31 ± 8.58	**0.007**	0.958	**0.001**	0.254
IL-8 mean ± SD (pg/ml)	49.84 ± 46.80	28.40 ± 23.26	6.45 ± 2.93	**<0.001**	0.492	**0.001**	0.463
VEGF mean ± SD (pg/ml)	26.51 ± 23.12	31.30 ± 43.11	18.51 ± 6.17	0.912	>0.99	0.822	0.627
VCAM mean ± SD (pg/ml)	1,661.19 ± 994.37	1,287.27 ± 574.85	188.68 ± 321.27	**<0.001**	0.789	**<0.001**	**0.004**

*AH, Aqueous humor; RV, Rubella virus; CMV, Cytomegalovirus; FUS, Fuchs uveitis syndrome; IL-10, Interleukin-10; IL-6, Interleukin-6; IL-8, Interleukin-8; VEGF, Vascular endothelial cell growth factor; VCAM, Vascular cell adhesion molecules; SD, Standard deviation*.

† Kruskal–Wallis H-test;

* *Bonferroni post-hoc test*.

*Bold values indicate P < 0.05*.

RV-IgG in the AH of the patients in the RV FUS group was significantly increased, and correspondingly, CMV-IgG in the AH of the patients in the CMV FUS group was also significantly increased. All the FUS patients showed positive results of GWC, with 24 RV-infected eyes showed positive RV GWC and 8 CMV-infected eyes showed positive results for CMV GWC. Although AH VZV-IgG concentration in the CMV FUS patients was higher than that in the RV FUS patients, the VZV-IgG concentration in AH was only slightly elevated in the CMV FUS patients and had negative GWC for VZV (Table 4).

**Table 4:** Virus-specific antibody in the AH of the patients with RV FUS and CMV FUS.

	**RV FUS**	**CMV FUS**	***P*-value^†^**
RV-IgG mean ± SD	46.16 ± 27.17	0.24 ± 0.40	**<0.001**
CMV-IgG mean ± SD	3.05 ± 1.96	52.08 ± 28.74	**<0.001**
HSV-IgG mean ± SD	0.52 ± 1.91	1.20 ± 1.84	0.068
VZV-IgG mean ± SD	0.04 ± 0.08	0.38 ± 0.61	**0.002**
EBV-IgG mean ± SD	0.16 ± 0.33	0.93 ± 1.63	0.097

*AH, Aqueous humor; FUS, Fuchs uveitis syndrome; RV, Rubella virus; CMV, Cytomegalovirus; HSV, Herpes Simplex Virus; VZV, Varicella Zoster Virus; EBV, Epstein-Barr virus; IgG, Immunoglobulin G; SD, Standard deviation*.

† *Mann–Whitney U-test*.

*Bold values indicate P < 0.05*.

## Discussion

In this study, we retrospectively analyzed the clinical characteristics and AH analysis results of FUS caused by two of the most common viral infections. RV is the main etiologic agent in Europe (90–100%) and the United States (3, 7, 10, 14, 15). In contrast, CMV has been reported to be a major cause of FUS in East Asia (5, 11). In this retrospective study, 8 (25%) of the 32 FUS patients were infected with CMV. The percentage of CMV-associated FUS in the present study is similar to the proportion of CMV infection found for FUS in Asians in previous studies (4, 5, 11). In agreement with previous reports, the FUS patients in both the RV and CMV groups in this study showed initial symptoms in their thirties or forties, with no gender predilection, and with blurring of vision or evaluation of floaters as the most frequently observed symptoms. Ocular manifestations include uniformly-distributed fine stellate KPs, mild anterior chamber inflammation, iris depigmentation or heterochromia, absence of posterior synechiae, variable vitreous inflammation and complicated cataract and glaucoma. Although FUS in immunocompetent patients caused by different viral etiologies (including RV and CMV) have similar clinical presentation, there are several differences that may support one viral etiology over the other.

In this study, clinical features that favor the diagnosis of CMV-associated FUS over RV-associated FUS include older age at disease onset and diagnosis, more severe visual impairment at the onset of disease, relatively poor visual prognosis, higher IOP during the course of the disease, higher incidence of glaucomatous optic neuropathy and lower grade vitreous inflammation.

The age at disease onset can be an important clue. The results in this study are consistent with the previous finding that older patients with FUS were more likely to have a CMV infection (11). Research has shown that CMV infects CD34+ myeloid progenitor cells during primoinfection. This enables CMV reactivation in the case of intercurrent infection. CMV can reactivate and shed on and off throughout life, particularly during intercurrent illness as circulating monocytes containing latent CMV are carried to sites of inflammation (5). The intraocular inflammation of FUS may be the result of local CMV reactivation in the anterior segment or may emerge from macrophage or dendritic cell activation (16). In addition, this study also found that the age at the diagnosis of FUS in the CMV group was significantly older than that in the RV group. As RV is the leading cause in Western populations, the features originally described by Fuchs can presumably describe the RV-induced FUS. CMV was not confirmed as a cause of FUS until recently when it was reported more frequently in Asia (17). The significant delay in diagnosis of CMV FUS might be caused by the lack of awareness of the characteristic clinical characteristics of this disease.

The severity of visual impairment at the onset of disease and the visual prognosis can also help in making the diagnosis clinically. A VA worse than 0.1 developed in none of the RV FUS patients, which is consistent with previously reported data (18). However, CMV FUS patients had more severe VA impairment than RV patients at both the first presentation and the final follow-up. One possible reason is that the ocular manifestations of this syndrome vary depending on the etiology. Another possible explanation is that patients in the CMV group were diagnosed later and had a higher rate of misdiagnosis, which means that the patients had a longer course of disease. All of these factors may contribute to poor vision in patients with CMV FUS.

The morphology and distribution of KPs are also clues. Although CMV and RV manifest as similar KPs, the KPs in RV FUS do not become pigmented, and in this study, the pigment KPs were observed in one of CMV FUS patient. Additionally, medium-sized KPs distributed in a ring pattern in coin-shaped lesions were observed in another CMV FUS patient. As mentioned in a previous study (17), the presence of pigment KPs or KPs distributed in a ring pattern in coin-shaped lesions serves as a clue that this condition may be of CMV rather than RV infection.

Previous studies showed that vitritis is very common in RV-associated uveitis and might be a salient clinical feature (6). Vitritis existing in RV-positive patients is relatively severe, with 15% of RV-affected eyes requiring vitrectomy due to vitreous opacities (6). By contrast, vitreous inflammation is mild or absent in CMV-associated FUS (19). CMV infection in immunocompetent individuals is mostly confined to the anterior segment (3). This study also found that RV-associated FUS tends to manifest as more severe vitreous inflammation than CMV-associated FUS. The severity of vitreous inflammation can also be used clinically to identify FUS caused by two groups of different etiologies.

The most common complication of FUS is the complicated cataract and the development of glaucomatous optic neuropathy, and glaucoma is the most serious vision-threatening complication. In this study, a chronically elevated IOP is a typical FUS feature present in most cases. The maximum IOP of CMV FUS was higher compared to that in the eyes with RV FUS, and the CMV group had a significantly higher proportion of developing glaucoma. Previous studies reported that CMV-associated FUS often necessitate long-term anti-glaucoma medications to control IOP (20) and glaucomatous optic neuropathy affects 36% of eyes (11). The results of this study also suggest that patients with CMV-associated FUS ought to be followed up closely for any significant increase in IOP or visual field defects.

In this study, we also analyzed the mainly inflammatory cytokines of the AH samples of those patients. The results of the analysis of inflammatory cytokines in the AH were consistent with the observed clinical manifestations. Moreover, inflammatory cytokines in AH were slightly elevated in FUS patients. These findings are consistent with the relatively mild AC inflammation in FUS. The comparison between the two FUS groups showed similar results, since both groups showed mild intraocular inflammation and mild elevation of cytokines in AH.

Since no cure is available for FUS, the exact etiology diagnosis may be considered not crucial, as it requires intraocular fluid sampling. Nonetheless, it is believed that an exact etiology diagnosis is important for improving the management and counseling of patients. Because each virus is treated differently, it is important to have a specific etiology for diagnosis. In the treatment of CMV infections, a study found that ganciclovir topical ointment is effective in preventing relapses of CMV-associated uveitis (19, 21), whereas oral valganciclovir appears to be more effective in acute episodes (22, 23). Another study showed that oral valganciclovir is effective in treating acute symptoms and in reducing the frequency of disease recurrence (24). It was also reported that if anti-CMV therapy can effectively control cytomegalovirus replication, anti-glaucoma surgery is rarely required (5). For the treatment of RV, no specific therapeutics has been suggested, and the reason is simple: no effective anti-rubella treatment exists. In patients with CMV infection, antiviral therapy could clear the viral load, assist the IOP control, and preserve the corneal endothelium (25). However, antiviral therapy does not have these effects on RV infection. The present study also found differences in the prognosis of FUS visual acuity and the risk of developing glaucoma due to infection with different viruses. Early diagnosis and appropriate treatment could help to prevent serious ocular complications. With the correct diagnosis of FUS, the patients no longer need unnecessary corticosteroids and other immunosuppressive therapies and can receive reliable information on potential future complications and the need for follow-up.

Although this study found differences in the clinical manifestations of FUS caused by the two different viruses, clinical examination was not sensitive enough in most cases to identify the causative virus. Firstly, as the ocular manifestations of the two groups are varied and may overlap considerably, it is difficult to make an accurate diagnosis of viral FUS on the basis of its clinical manifestations only. Secondly, there are individual differences in clinical features among different patients. The possibility that some patients with atypical signs were misdiagnosed as other types of uveitis cannot be excluded. For example, although regarded as a sign of granulomatous uveitis, Busacca nodules can also be seen in a few FUS patients, which may result in misdiagnosis as granulomatous uveitis. Patients may also develop floaters due to vitritis and may be misdiagnosed as intermediate uveitis. Thirdly, the clinical manifestations of different stages of the disease are also different. Yang et al. (26) reported that the morphology of KPs might change in appearance over time. Finally, the primary clinical concern and the previous treatment process may also affect the final accurate diagnosis of the disease. FUS is often misdiagnosed and patients are treated with topical, systemic, and peribulbar steroids. In this study, the diagnosis of FUS was made in an average of 1.9 years after symptoms onset and all the FUS patients had been treated with topical steroid before their referral.

As a result, although clinical manifestations are the most fundamental basis for the diagnosis of disease, clinical manifestations may not always be sufficient to differentiate between various forms of viral FUS. Laboratory AH analyses may help identify (and subsequently select treatment strategies) these entities. A correct and prompt diagnosis helps to avoid unnecessary and potentially dangerous therapies. PCR-based analyses of AH samples can help identify minimal amounts of viral DNA and realize rapid confirmation of the diagnosis. GWC calculations help to determine the pathogen-specific production of intraocular antibodies to determine whether an infection has occurred. (27). Real-time PCR also can help to quantify the viral load as an indicator of the severity of the infection. However, all diagnostic tests have their limitations, and the AH assessment is no exception. In most cases, a positive AH assessment for RV or CMV can confirm a diagnosis of RV- associated or CMV-associated uveitis, and therefore the critical appraisal of the exact laboratory results and clinical manifestations is needed. Similarly, a negative PCR result does not rule out virus infection; this may be to the result of the low intraocular viral load, the limited volume of AH samples, the short-lived phase of DNA release that may have been missed during the time of aqueous sampling because of delayed presentation or testing (27). The sensitivity of PCR and the limitations of GWC laboratory techniques, as well as the leakage through a damaged blood-retina barrier, may have affected the results of laboratory analysis. Additionally, the laboratory analysis results of AH vary with different courses of the disease. PCR is usually positive at onset and/or during early reactivation, and GWC analysis is likely to take as many as 2 weeks to become positive in the acute phase but remains positive for longer time frames (27). GWC is more useful when patients have low levels of DNA after the acute episode; and PCR is more helpful than GWC analysis in patients who present symptoms during the acute episode and immunocompromised patients with aberrant antibody synthesis (17). As misdiagnosis and delayed diagnosis are common in FUS patients, it is important to select the appropriate test method and interpret the test results correctly. In this study, FUS was diagnosed previously in only 31.3% of the patients by the referring ophthalmologists and the diagnosis of FUS was made in an average of 1.9 years after symptoms onset. All the patients had been treated with topical steroid before. Aqueous tapping should be done in patients who are referred for management of FUS in order to determine whether to administer antiviral medication targeting CMV. The choice of AH laboratory analysis depends on the patient's clinical symptoms, the individual's immune status, the chronicity of infection and the time of AH sampling. Therefore, the accurate etiological diagnosis of FUS and the formulation of appropriate treatment strategies require a comprehensive analysis of clinical manifestations, a careful selection of appropriate laboratory methods and a correct interpretation of results.

Undeniably, this study has its limitations. First, the number of the FUS patients enrolled in the study was not sufficient and the follow-up time was not long enough. Second, the retrospective nature of the study made it impossible to accurately compare some of the important ophthalmologic findings, such as endothelial cell counts. As RV is generally believed to be the primary cause of FUS, endothelial cell examination and follow-up are not included in the routine FUS screening. This also suggests that CMV FUS patients should be monitored for damage of corneal endothelium after the accurate etiological diagnosis is confirmed by laboratory analysis of AH, while RV FUS patients should be routinely examined and followed up. Third, this study was conducted only among immunocompetent patients and did not consider patients with immunodeficiency. Fourth, as this clinic is a tertiary referral center, the referral bias of samples is unavoidable. Further long-term prospective multicenter studies are required to investigate detailed clinical characteristics and disease courses of FUS patients with different etiological infections.

Compared with RV FUS, CMV FUS had older age at disease onset and diagnosis, more severe visual impairment at the onset of disease, relatively poorer visual prognosis, higher IOP during the course of the disease, higher incidence of glaucomatous optic neuropathy, and lower grade vitreous inflammation. The observed clinical differences between RV FUS and CMV FUS may facilitate the identification of the pathogeny of viral FUS. However, since each virus has variable clinical representations, and different viruses may also display overlapping manifestations, the preferred etiology-confirming methods are PCR or GWC assay of AH samples. Thus, comprehensive assessment of clinical characteristics and laboratory analysis of AH are preferred to confirm a specific etiological diagnosis, determine therapeutic strategies, and monitor the response to therapy.

## Data Availability Statement

The original contributions presented in the study are included in the article/supplementary materials, further inquiries can be directed to the corresponding author/s.

## Ethics Statement

The institutional review board approved this retrospective study. AH and peripheral blood (PB) samples of all the patients were obtained only after informed consent and Ethics Committee approval. All procedures were performed according to the tenets of the Declaration of Helsinki.

## Author Contributions

Design and conduct of the study by HK and YT. Collection, management, analysis and interpretation of the data by HK, HB, YS, JF, WY, YH, HW, and XH. Preparation of the manuscript by HK and YT. Review and final approval of the manuscript by all the authors.

## Conflict of Interest

The authors declare that the research was conducted in the absence of any commercial or financial relationships that could be construed as a potential conflict of interest.
